# Workplace social support, mental health and work functioning among young workers

**DOI:** 10.1093/eurpub/ckac129.439

**Published:** 2022-10-25

**Authors:** S de Groot, K Veldman, BC Amick, U Bültmann

**Affiliations:** 1 Health Sciences, Community and Occupational Medicine, University of Groningen, Groningen, Netherlands; 2 Health Sciences, Community and Occupational Medicine, University Medical Center Groningen, Groningen, Netherlands; 3 Winthrop Rockefeller Cancer Institute, University of Arkansas for Medical Sciences, Little Rock, USA

## Abstract

**Background:**

Young adults with a history of mental health problems (MHPs) report lower work functioning (WF) compared to their peers without a history of MHPs. The identification of modifiable, protective workplace factors, such as workplace social support, is needed to increase WF.We examined the moderating role of workplace social support from supervisors and co-workers in the prospective association between MHP trajectories from childhood to young adulthood and WF among young adults.

**Methods:**

The most recent 2019/2020 data of N = 861 young workers, participating in the 18-year follow-up Dutch TRAILS (TRacking Adolescents’ Individual Lives Survey) cohort study, was used. MHP trajectories for internalising and externalising problems included measurements at ages 11, 13, 16, 19, 22 and 26. Supervisor and co-worker social support were measured at age 29. WF was assessed at age 29. Logistic regression analyses were conducted to examine the moderating role of workplace social support in the association between MHP trajectories and WF.

**Results:**

Four trajectories were identified for both internalising and externalising problems. Young adults with high-stable MHP trajectories reported more often low WF (ORs 3.73 (95% CI 2.28-6.12) and 2.88 (1.78-4.65) for internalising and externalising problems respectively) than those with low-stable trajectories. Higher supervisor and co-worker social support were associated with a lower odds for low work functioning (adjusted ORs ranging from 0.67 (0.54-0.83) to 0.84 (0.71-1.00)). No moderating effect of workplace social support was found for the association between MHP trajectories and WF.

**Conclusions:**

Both supervisor and co-worker support were shown to be important for all young workers, regardless their history of mental health problems. Occupational health professionals should create awareness among employers and employees that workplace social support is beneficial for young adults’ work functioning.

**Key messages:**

• Both supervisor and co-worker social support are important for all young workers’ work functioning, regardless of their history of mental health problems.

• Creating awareness of the impact of workplace social support on young adults’ work functioning among employers and employees should be a priority area for occupational health practice and policy.

